# Supplementary material for: Molecularly Designed Ion-Imprinted Nanoparticles for Real-Time Sensing of Cu(II) Ions Using Quartz Crystal Microbalance
Source: Biomimetics (Basel). 2022 Nov 5;7(4):191. doi: 10.3390/biomimetics7040191 (PMC9680276; doi:10.3390/biomimetics7040191)
Supplement: Supplementary file 1 [file biomimetics-07-00191-s001.zip › SupplementaryData1.pdf]

Supplementary Data 1
for

Molecularly Designed Ion Imprinted Nanoparticles for Real-Time Sensing of Cu(II) Ions using Quartz Crystal Microbalance

a) MAH structure

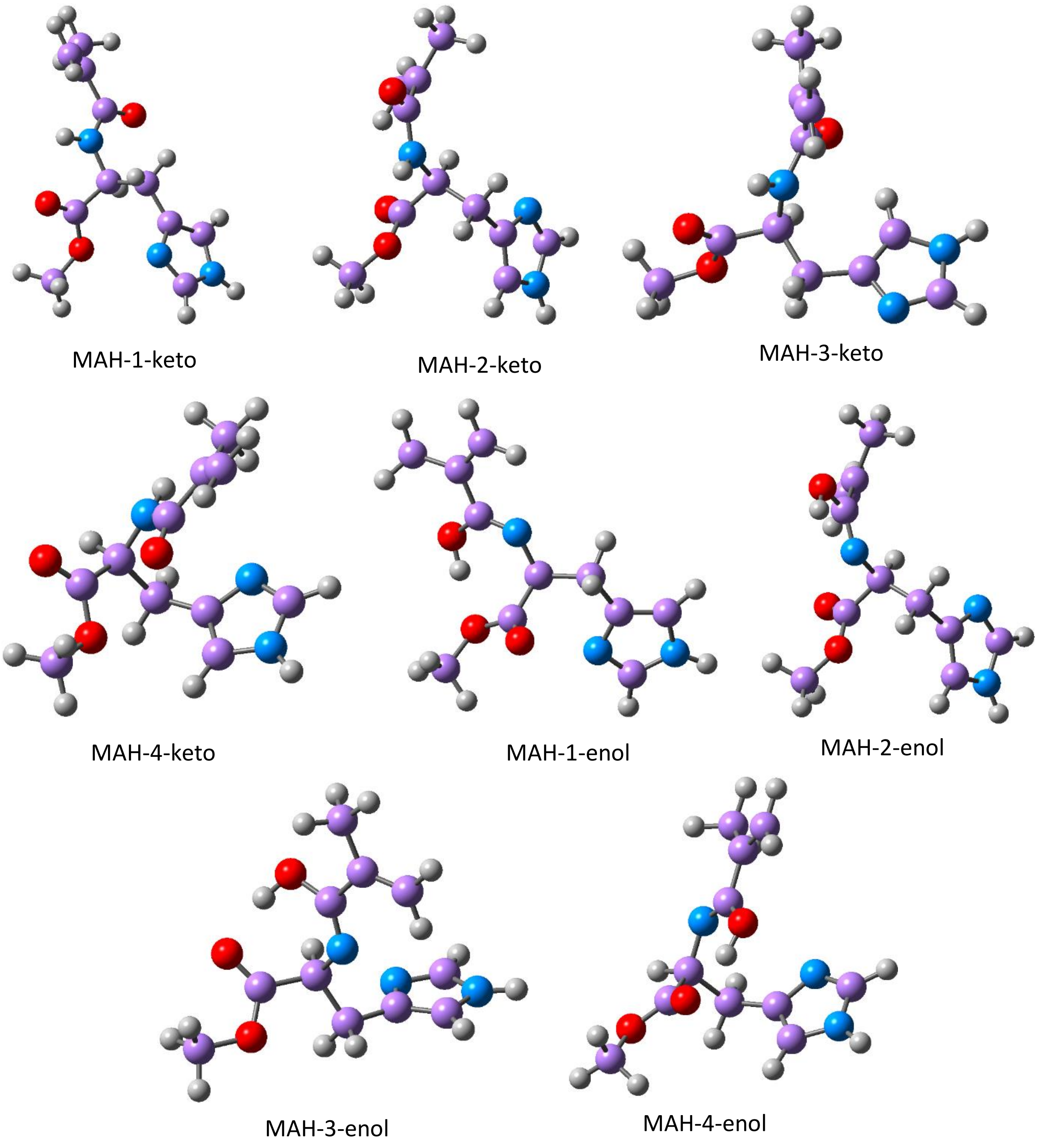

Figure SD1-1. The optimized geometries of the keto and enol conformers of MAH structure.

Table SD1-1. The total and relative energies of keto and enol tautomers of MAH structure

BP86/6-31G(d,p)	Total Energy (hartree)	Relative Energy (kcal/mol)
MAH-1-keto	-818,1591729	0,63
MAH-2-keto	-818.1500523	6.35

MAH-3-keto	-818.1601783	0.00
MAH-4-keto	-818.1483918	7.40
MAH-1-enol	-818.1344719	16.13
MAH-2-enol	-818.1315501	17.96
MAH-3-enol	-818.1388452	13.39
MAH-4-enol	-818.1369506	14.58

b) Cu(II)-MAH aqua complexes

Table SD1-2. The total, binding and relative energies of four-fold structures of Cu(II)-MAH complexes

Four-fold structures	Total Energy (hartree)	BE	relative E kcal/mol
MAH-Cu-2N-2aq-keto	-1166.756902	-0.6228299	20.71
MAH-Cu-2N-2aq-enol	-1166.760178	-0.6474388	5.26
MAH-Cu-NO-2aq-enol	-1166.755198	-0.6424590	8.39
MAH-Cu-OO-2aq-keto	-1166.769448	-0.6353752	12.83
MAH-Cu-OO-2aq-enol	-1166.725683	-0.6129438	26.91
MAH-Cu-RingN-3aq-keto	-1243.205197	-0.6515438	2.69
MAH-Cu-RingN-3aq-enol	-1243.188149	-0.6558289	0.00
MAH-Cu-RingNO-2aq-keto	-1166.772855	-0.6387827	10.70
MAH-Cu-ONO-aq-enol	-1090.296100	-0.6029413	33.19
Cu(II)	-195.1347327		
aqua	-76.4195807		

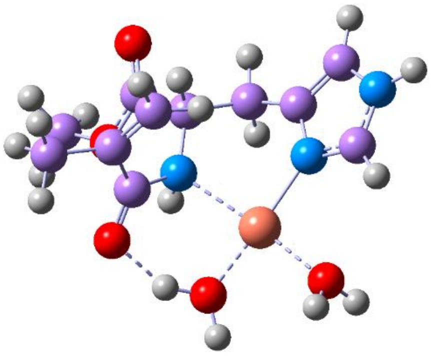

MAH-Cu-2N-2aq-keto

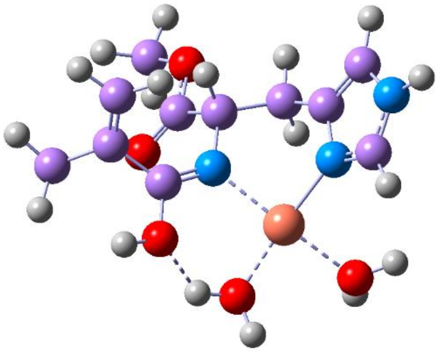

MAH-Cu-2N-2aq-enol

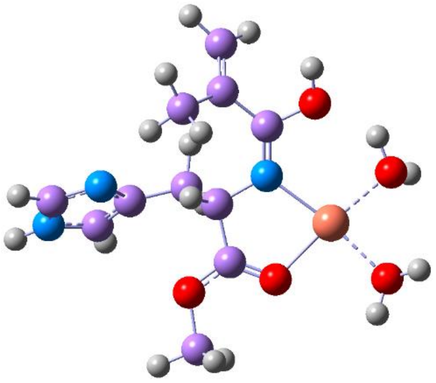

MAH-Cu-NO-2aq-enol

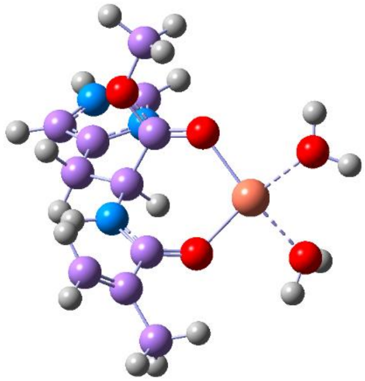

MAH-Cu-OO-2aq-keto

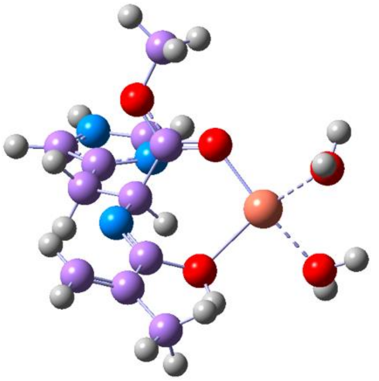

MAH-Cu-OO-2aq-enol

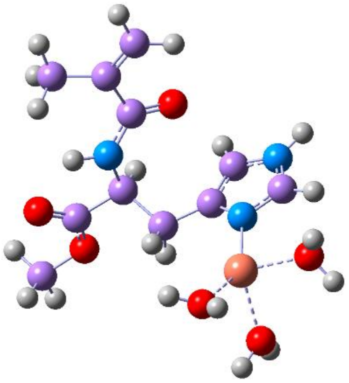

MAH-Cu-Ring-N-3aq-keto

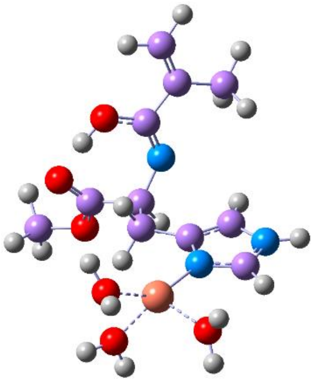

MAH-Cu-Ring-N-3aq-enol

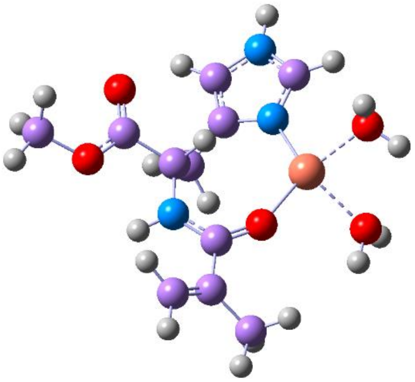

MAH-Cu-RingNO-2aq-keto

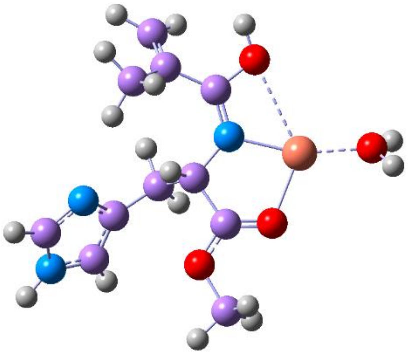

MAH-Cu-ONO-aq-enol

Figure SD1-2. The optimized geometries of four-fold structures of Cu(II)-MAH complexes

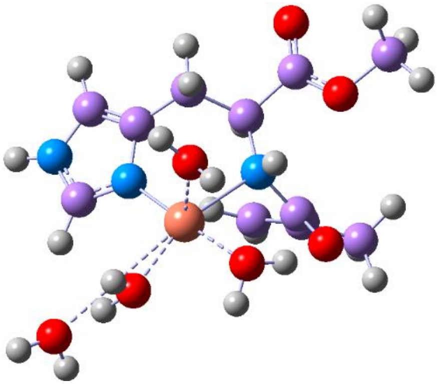

MAH-Cu-2N-4aq-keto

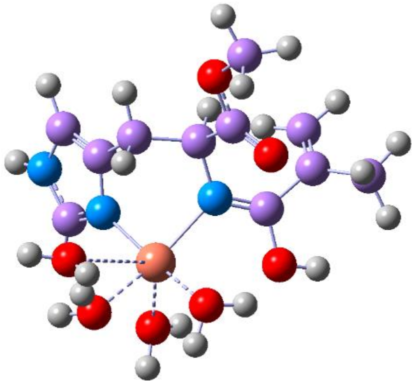

MAH-Cu-2N-4aq-enol

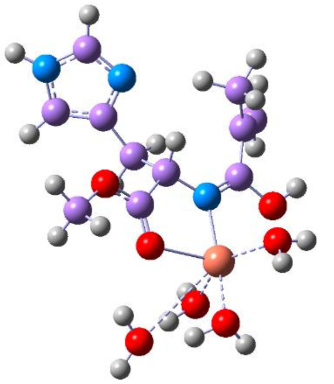

MAH-Cu-NO-4aq-enol

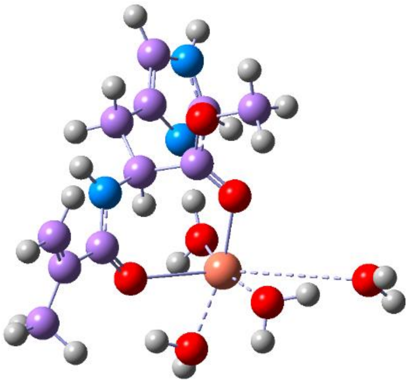

MAH-Cu-OO-4aq-keto

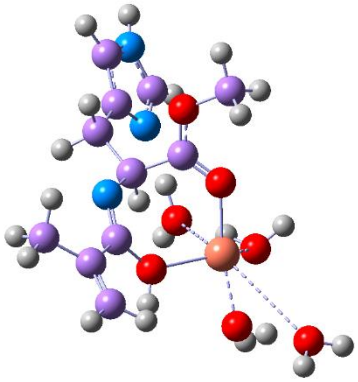

MAH-Cu-OO-4aq-enol

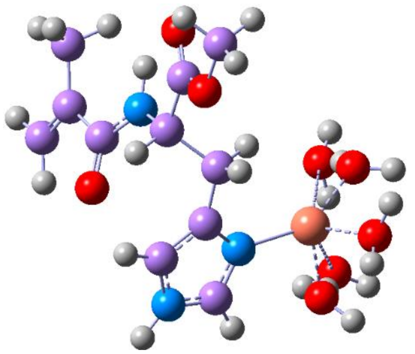

MAH-Cu-RingN-5aq-keto

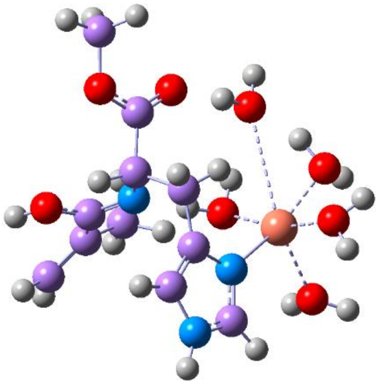

MAH-Cu-RingN-5aq-enol

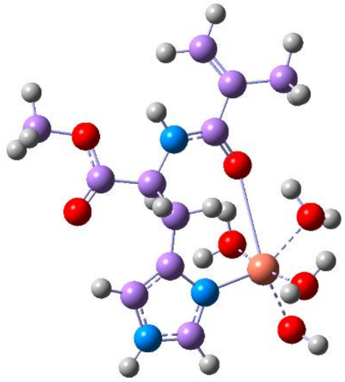

MAH-Cu-RingNO-4aq-keto

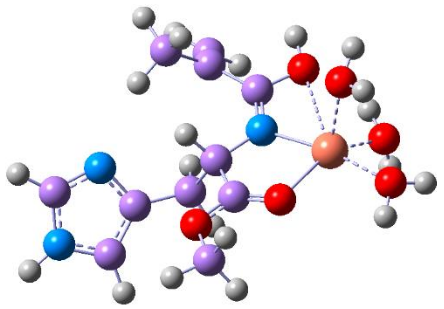

MAH-Cu-ONO-3aq-enol

Figure SD1-3. The optimized geometries of six-fold structures of Cu(II)-MAH complexes

Table SD1-3. The total, binding and relative energies of six-fold structures of Cu(II)-MAH complexes

Six-fold structures	Total Energy	BE	relative E kcal/mol
MAH-Cu-2N-4aq-keto	-1319.666417	-0.6931828	35.59
MAH-Cu-2N-4aq-enol	-1319.66494	-0.7130388	23.13
MAH-Cu-NO-4aq-enol	-1319.65187	-0.6999693	31.34
MAH-Cu-OO-4aq-keto	-1319.68437	-0.711136	24.33
MAH-Cu-OO-4aq-enol	-1319.645785	-0.6938844	35.15
MAH-Cu-RingN-5aq-keto	-1396.112024	-0.7192094	19.26
MAH-Cu-RingN-5aq-enol	-1396.121387	-0.7499056	0.00
MAH-Cu-RingNO-4aq-keto	-1319.676061	-0.7028273	29.54
MAH-Cu-ONO-3aq-enol	-1243.203379	-0.6710592	49.48

c) ***Cu(II)-(MAH)$_2$ aqua complexes***

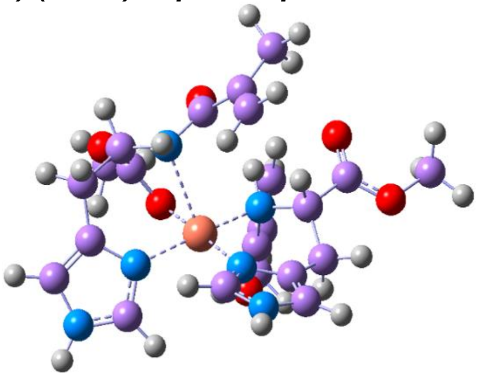

2MAH-Cu-2N-keto

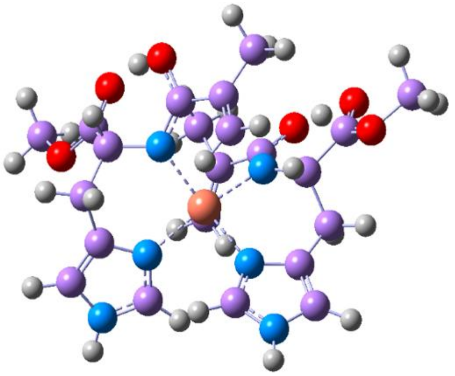

2MAH-Cu-2N-enol

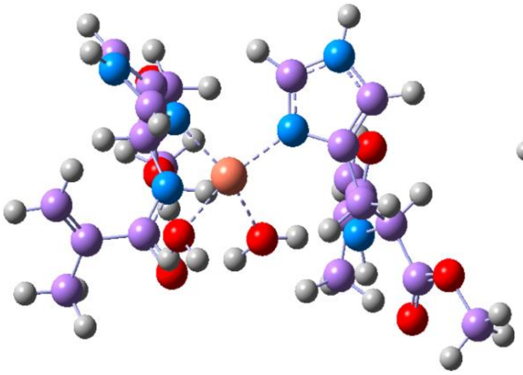

2MAH-Cu-RingN-2aq-

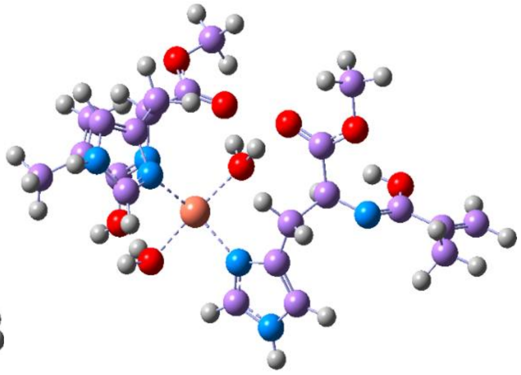

2MAH-Cu-RingN-2aq-

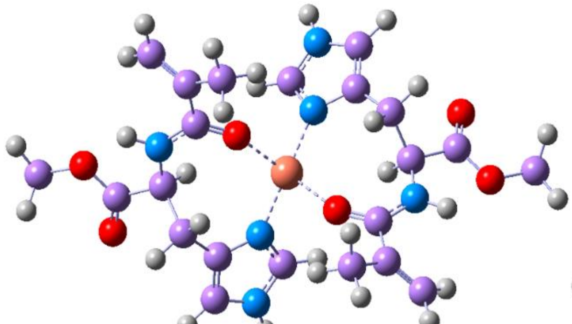

2MAH-Cu-RingNO-keto

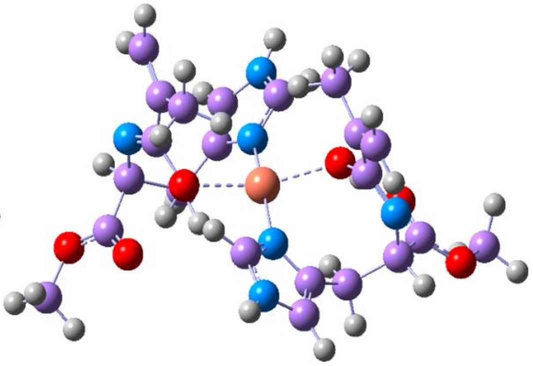

2MAH-Cu-RingNO-enol

Figure SD1-4. The optimized geometries of four-fold structures of Cu(II)-(MAH)$_2$ complexes

Table SD1-4. The total, binding and relative energies of four-fold structures of Cu(II)-$(MAH)_2$ complexes

Four-fold structures	Total Energy (hartree)	Binding Energy hartree	Relative Energy kcal/mol
2MAH-Cu-2N-keto	-1832.112041	-0.6569512	34.18
2MAH-Cu-2N-enol	-1832.105943	-0.6935200	11.23
2MAH-Cu-RingN-2aq-keto	-1984.988207	-0.6939566	10.96
2MAH-Cu-RingN-2aq-enol	-1984.963007	-0.7114229	0.00
2MAH-Cu-RingNO-keto	-1832.121363	-0.6662735	28.33
2MAH-Cu-RingNO-enol	-1832.069192	-0.6567690	34.30

Table SD1-5. The total, binding and relative energies of four-fold structures of Cu(II)-$(MAH)_2$ complexes

Six-fold structures	Total Energy (hartree)	Binding Energy hartree	Relative Energy kcal/mol
2MAH-Cu-2N-2aq-keto	-1984.997084	-0.7028335	39.34
2MAH-Cu-2N-2aq-enol	-1984.977263	-0.7256784	25.00
2MAH-Cu-RingN-4aq-keto	-2137.89126	-0.7578476	4.82
2MAH-Cu-RingN-4aq-enol	-2137.856273	-0.7655268	0.00
2MAH-Cu-RingNO-2aq-keto	-1985.015234	-0.7209830	27.95
2MAH-Cu-RingNO-2aq-enol	-1984.952924	-0.7013391	40.28

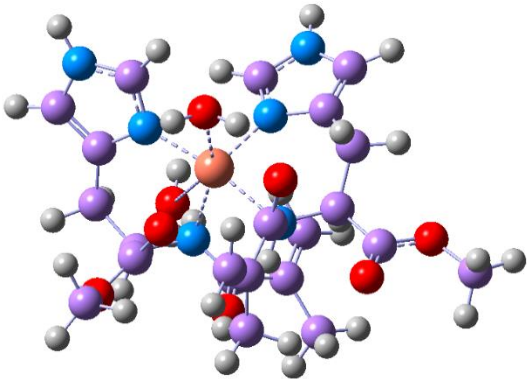

2MAH-Cu-2N-2aq-keto

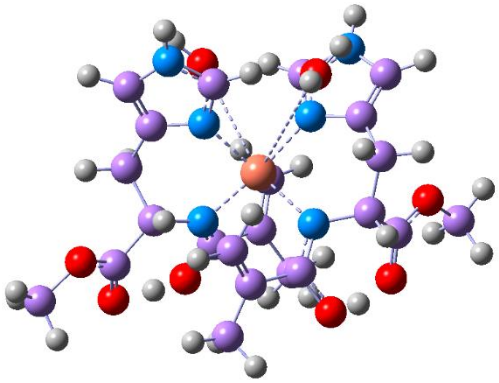

2MAH-Cu-2N-2aq-enol

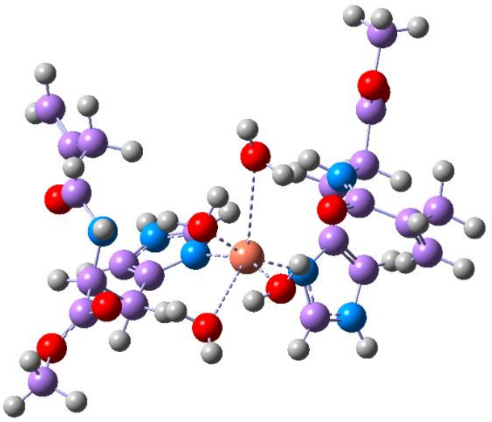

2MAH-Cu-RingN-4aq-keto

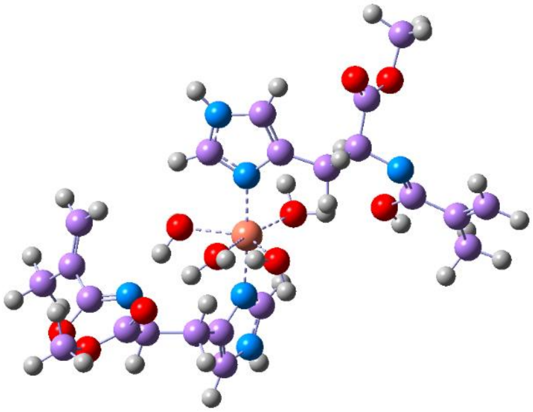

2MAH-Cu-RingN-4aq-enol

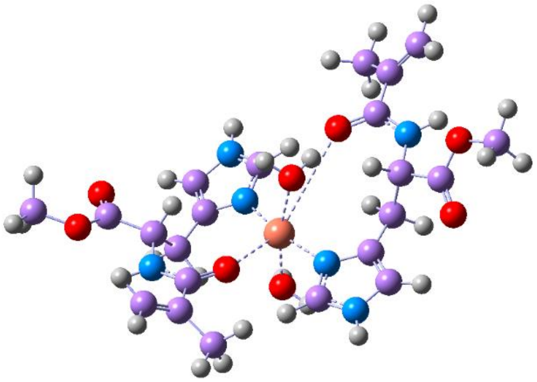

2MAH-Cu-RingNO-2aq-keto

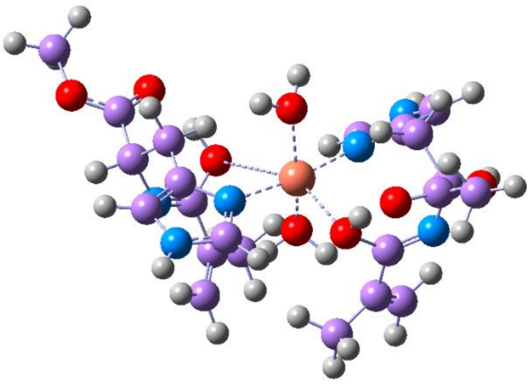

2MAH-Cu-RingNO-2aq-enol

Figure SD1-5. The optimized geometries of six-fold structures of Cu(II)-$(MAH)_2$ complexes.
