# Supplementary material for: Molecularly Designed Ion-Imprinted Nanoparticles for Real-Time Sensing of Cu(II) Ions Using Quartz Crystal Microbalance
Source: Biomimetics (Basel). 2022 Nov 5;7(4):191. doi: 10.3390/biomimetics7040191 (PMC9680276; doi:10.3390/biomimetics7040191)
Supplement: Supplementary file 1 [file biomimetics-07-00191-s001.zip › SupplementaryData2.pdf]

Supplementary Data 2

for

Molecularly Designed Ion Imprinted Nanoparticles for Real-Time Sensing of Cu(II) Ions using Quartz Crystal Microbalance

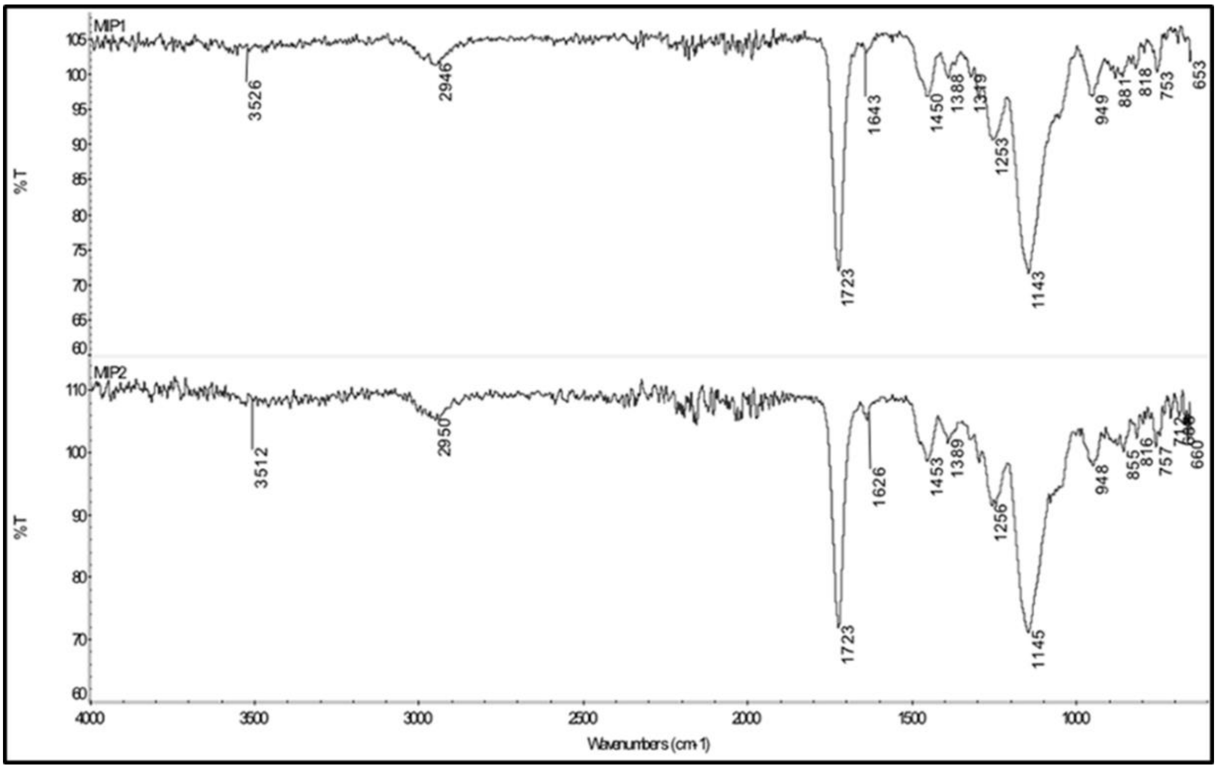

Figure SD2-1. FTIR spectra of Cu(II)-MIP1 (upper) and MIP2 (lower) nanoparticles.
